# Proteins Derived from the Dairy Losses and By-Products as Raw Materials for Non-Food Applications

**DOI:** 10.3390/foods10010135

**Published:** 2021-01-10

**Authors:** Catarina Costa, Nuno G. Azoia, Lorena Coelho, Ricardo Freixo, Patrícia Batista, Manuela Pintado

**Affiliations:** 1 Centre of Nanotechnology and Smart Materials (CeNTI), Rua Fernando Mesquita, 2785, 4760-034 Vila Nova de Famalicão, Portugal; cdcosta@centi.pt (C.C.); nazoia@centi.pt (N.G.A.); lcoelho@centi.pt (L.C.); 2 CBQF-Centro de Biotecnologia e Química Fina-Laboratório Associado, Escola Superior de Biotecnologia, Universidade Católica Portuguesa, Rua Diogo Botelho 1327, 4169-005 Porto, Portugal; ricardofreixo1@hotmail.com (R.F.); pbatista@porto.ucp.pt (P.B.)

**Keywords:** whey protein, casein, textile fibre, textile finishing, functional properties

## Abstract

The disposal of a high volume of waste-containing proteins is becoming increasingly challenging in a society that is aware of what is happening in the environment. The dairy industry generates several by-products that contain vast amounts of compounds, including proteins that are of industrial importance and for which new uses are being sought. This article provides a comprehensive review of the potential of the valorisation of proteins that can be recovered by chemical and/or physical processes from protein-containing milk by-products or milk surplus, particularly whey proteins or caseins. Whey proteins and casein characteristics, and applications in non-food industries, with special emphasis on the textile industry, packaging and biomedical, are reported in this review, in order to provide knowledge and raise awareness of the sustainability of these proteins to potentiate new opportunities in a circular economy context.

## 1. Introduction

In the dairy industry, milk processing generates several by-products that could represent an environmental problem if discarded incorrectly. The cheese industry generates the most abundant and pollutant by-product: the whey. The large amount produced, although a problem for the dairy industry, may also represent an opportunity for food, but also for other industries, which are seeking new ways of using these by-products as raw materials for several applications. To valorise the protein derived from the dairy by-products, various technological approaches have been used to transform this by-product into value-added products. During the last 3 decades the whey have been converted into products such as whey powder, whey protein concentrates or isolates, whey permeate, bioethanol, biopolymers, hydrogen and methane, among others. Therefore, it is not a by-product, but is rather seen as a valuable co-product of cheese making and casein production. The whey protein represents 20% of total milk protein (β-lactoglobulin, α-lactalbumin, serum albumin and immunoglobulins) and it is composed by amino acids (branched and essential) and functional peptides with different bioactive properties, including antioxidant activity. Despites these by-products being generated by the regular milk processing, sometimes there are large amounts of discarded milk that do not comply with the food industry safety regulations, or even production surplus. This discarded milk is rich in casein, whey protein and lactose, and also has huge potential for use as raw materials in other applications.

Most of the applications have been performed in the development of new food ingredients and products. However, the new context of the circular economy, has led to the generation of industrial synergies, where those proteins have been innovatively used in new sectors, where new products have been created with marked competitive value, namely packaging, textile and biomedical. However, there are no reviews including this new vision of milk protein applications in the context of the sustainability and diversity of non-food applications.

## 2. Compositional Value and Properties of Milk Derivatives

Milk is a colloidal dispersion consisting of water, lipids, proteins, lactose and minerals [1]. It also contains several minor components, such as enzymes, organic acids, nitrogenous compounds and vitamins, as described in Table 1. The protein profile of bovine milk includes caseins, β-lactoglobulin, α-lactalbumin, serum albumin, immunoglobulins, lactoferrin and lactoperoxidase. The milk constituents are either present in a solution (lactose, salts and other minor substances) or organized into an emulsified state (fat globules) or dispersed colloidal particles. The caseins, a group of insoluble proteins stable as a micellar phase in milk, account for 80% of the protein content, and the other 20% are soluble proteins and usually called whey proteins [2]. The structure and assembly of the different milk constituents confer unique physicochemical properties to milk, which in turn define its behaviour during processing and dairy-product characteristics.

### 2.1. Casein and Caseinates

Caseins represent 80% of the total protein content of milk and can be easily recovered by isoelectric precipitation or enzymatic coagulation, typically using chymosin. In both cases, whey is obtained as a by-product [6]. Caseins are present in bovine milk as macromolecular aggregates, the main ones (α, β and κ) being different from each other in terms of phosphate content (10, 5 and 1 mol per mol of casein) [7]. Casein, when hydrolysed, may generate biologically active molecules, among them phosphorylated peptides, which exert an effect on the bioavailability of calcium and other highly anionic minerals [8,9].

Although most of the casein applications are in the food sector, there are several non-food-related applications of casein. Nowadays, casein glues are still used in labeling adhesives, in the bottling industry or in interior woodworking, as well as in paper finishing; however, the costs are higher [10].

The antioxidant activity of casein and its hydrolysates were determined in several studies. Among them, Sakanaka et al. (2005) suggested that the peptides obtained from the hydrolysis of casein can be effectively used as antioxidants against lipid oxidation in food systems, avoiding damage and increasing their shelf-life [7]. Rival, Fornaroli, Boeriu, and Wichers, (2001) have shown that caseins and casein-derived peptides had lipoxygenase-inhibitory properties, as well as enzymatic and non-enzymatic lipid peroxidation inhibitory properties [11]. Kitts (2005), has showed that a bioactive phosphopeptide derived from casein exhibited both primary and secondary antioxidant activity towards transition ferrous iron sequestering and direct free-radical-quenching activities in both aqueous and lipid emulsion systems [9].

### 2.2. Whey Derived Components

In a normal milk-processing plant, two different whey products can be obtained: sweet whey and acid whey. Sweet whey results from the production of some cheeses (cheddar, mozzarella and Swiss), where casein’s precipitation is promoted by enzymatic cleavage. The acid whey is obtained from the production of cheeses such as cottage cheese, cream cheese and Ricotta. In this case, casein’s precipitation is promoted by milk acidification. From both types of whey, whey powders can be obtained directly by water evaporation. The compositions of the two types of whey powders are described in Table 2 [12].

#### 2.2.1. Whey Protein Concentrates

Besides whey powders, different whey products can be obtained. The proteins in the whey can be concentrated and purified using membrane filtration systems [13], and after water removal it is possible to obtain whey protein concentrates (WPC), with protein contents ranging from 34% to about 85%. With protein contents higher than 90%, these powders are named whey protein isolates (WPI), a product with high-quality protein. Whey powder is normally used as animal feedstock or as medium for yeast and bacterial growth. Only a small part of it is used for human consumption due to its low protein content and high sugar concentration. On the other hand, those WPC and isolates (WPI) are highly valued for human consumption due to their composition, namely high protein concentration, low calories and low-fat content [14]. As dietary supplements, whey proteins are known to have several biological functions such as cancer prevention, and antibacterial and antifungal activities [6]. Whey proteins are also can be used as food ingredients, owing to some functional properties like good water solubility, high viscosity, emulsifying and gel formation ability [15].

#### 2.2.2. β-Lactoglobulin

β-Lactoglobulin (β-LG) is the most abundant whey protein of bovine milk, being absent from human milk. It is a globular protein that exists as a dimer on a pH scale between 5 and 7, due to electrostatic interactions. For pH values between 3.5 and 5.0, it exists in the form of an octamer or as a monomer at pH values below 3.5 [16]. Native β-LG possesses two disulphide bonds, with one free thiol group. The native conformation is sensitive to heat and pH value, and the free thiol group is of major significance in denaturation. The mechanism for heat denaturation consists of dimer dissociation followed by monomer unfolding to allow a faster thiol reactivity that can lead to disulphide interchange and aggregation, although noncovalent aggregation can also occur without the involvement of the thiol group [16].

It is generally accepted that β-LG dictates the water-holding characteristics (viscosity and gelation) of whey-protein-based ingredients. Lorenzen and Schrader (2006) showed the relationship between gelation properties of WPI an WPC, and concluded that these properties are higher in WPI, because of the higher content of β-lactoglobulin [17]. The strength of heat-induced gels prepared with WPI is higher than with WPC. Furthermore, WPI gels are more elastic than WPC gels.

#### 2.2.3. α-Lactoglobulin

The second most abundant whey protein is α-lactalbumin, a low-molecular-weight protein with 123 amino acid residues. Its primary function is to modulate the enzymatic activity of the lactose synthase, the enzyme responsible for lactose biosynthesis. α-Lactalbumin binds metal cations with a high affinity to calcium and several other ions. This binding, except Zn^2+^, strongly increases the stability of α-lactalbumin. α-lactalbumin is able to interact with hydrophobic peptides like melittin and with lipid membranes [18].

#### 2.2.4. Lactoferrin

Lactoferrin, or lactotransferrin, is a multifunctional 703-amino acid glycoprotein originally isolated from milk. The name resulted from a past classification as a major iron-binding protein in milk [19]. Lactoferrin displays a wide array of modes of action to execute its primary antimicrobial function. It presents great antimicrobial activity against bacterial, viral, and fungal in vitro [20]. Their antimicrobial action is executed by the intact molecule, and by their monoferric lobes or active peptides, which have an important function in the host defense against microbial disease. Lactoferrin presents several antimicrobial peptides that they are released upon the proteolysis of this molecule by various proteolytic enzymes. Moreover, in some cases, the peptides released in the enzymatic digestion of lactoferrin present a greater antimicrobial potential than the protein in its native form [20].

Lactoferrin may also have the biological function of protection against oxidative damage, due to the elimination of excess iron and other divalent metal ions that catalyze the unwanted formation of free radicals. The excessive formation of free radicals such as superoxides or hydroxyl radicals are among the main causes of skin damage. These damages are characterized by the appearance of wrinkles and other symptoms of aging [12].

Non-food uses of lactoferrin have been developed in recent years. For example, the antibacterial activity of lactoferrin has been utilized in toothpaste and mouthwash. An antibacterial mouthwash, for example, contains a combination of lactoferrin, lactoperoxidase and lysozyme [12].

#### 2.2.5. Lactoperoxidase

Lactoperoxidase is glycoprotein naturally present in raw milk, colostrum, saliva and other biological secretions, both human and animal [4]. This is the most abundant enzyme in milk, belonging to the peroxidase family, a group of enzymes found in nature that catalyzes the peroxidation of organic molecules. Lactoperoxidase plays an important role in the naturally occurring antibacterial system in milk known as the lactoperoxidase system (LPS). This system consists of three components, lactoperoxidase, thiocyanate and hydrogen peroxide (H_2_O_2_), and it is active only in the presence of all three components. Lactoperoxidase catalyses the oxidation of thiocyanate (SCN^−^) by H_2_O_2_ and obtains products with antibacterial properties [21]. The LPS can be employed as an antimicrobial pre-treatment to allow low-temperature thermal treatment of heat-sensitive foods and beverages, and the beneficial potential in the eradication of microorganisms from fresh food surfaces (e.g., meat) and in the preservation of cosmetic products has been reported in the literature [21]. Additionally, the LPS system can increase the shelf life of raw milk, having been investigated as an alternative to pasteurization in order to extend the quality of milk preservation, particularly in regions where refrigeration is not available, and has already been used in processed infant formulas, ice creams, creams, cheeses and whole eggs [4,22].

#### 2.2.6. Caseinomacropeptide

Caseinomacropeptide (CMP) is a phosphoglycopeptide, molecular weight of 7 kDa, produced by proteolysis of κ-casein by gastric pepsin, or during enzymatic coagulation of milk by chymosin, in which case it will dissolve into whey [3]. Some studies suggested that CMP exhibit different biological activities associated with microbiota control in the gastrointestinal tract [23]. Robitaille and Champagne (2014) evaluated the effect of pepsin-treated CMP on probiotics and concluded that it is effective in promoting the growth of all probiotic bacteria tested on milk [24].

### 2.3. Functional Properties of Milk Derivates

The total antioxidant capacity (hydrophilic plus lipophilic) of bovine milk and deproteinized milk, milk whey and its low molecular weight fractions from pasteurized and ultra-high temperature (UHT) treatments were evaluated by spectrophotometric methods (ABTS and FRAP) and electrochemical flow-injection amperometry (FIAmp) [25]. Analyses of the whole milk, whey and deproteinized milk showed that the casein fractions were the relevant constitute with antioxidant properties of whole milk, whereas the albumin protein was the relevant constitute with antioxidant properties of whey protein. For the deproteinized milk, the hydrophilic antioxidant compounds (vitamin C and uric acid) were highlighted as the constitutes responsible for antioxidant properties. Significant differences in total antioxidant properties were found between whey and deproteinized milk.

Corrêa et al. (2014) evaluated the antioxidant activity of sheep cheese whey (SCW) hydrolysates obtained by enzymatic treatment, which allowed the release of bioactive peptides [26]. The antioxidant activity of hydrolysates, obtained by the method of elimination of the radical ABTS (2,2-azino-bis-(3-ethylbenzothiazoline)-6-sulfonic acid), increased 3.2-fold from 0 h (15.9%) to 6 h of hydrolysis (51.3%). The SCW hydrolysates, presenting antioxidant and ACE-inhibitory properties, might be potentially employed to retard lipid oxidation and deterioration of foods, and as functional ingredients.

In order to find out if hydrolysates obtained from whey can be used as a source of antioxidants Dryáková and collaborators (2010) studied hydrolysis with four microbial proteases (Alcalase, Flavourzyme, Neutrase and Protamex) [27]. The hydrolyzed samples were tested by different methods, including the ABTS radical elimination method. Antioxidant properties were increased by hydrolysis, and the hydrolysates obtained by the protease Alcalase were considered the most effective antioxidants. It is known that milk and certain derived fractions are effective in absorbing odors, due to their composition of proteins and lipids. Therefore, its incorporation in textiles can also allow the exploitation of this functionality and its development, being a pioneering approach in the state and a cutting-edge technology for textile application [28]. In particular, lactose is described by its ability to retain odors, absorbing them on its surface as the crystals form [29].

## 3. Application of the Proteins Derived from the Dairy Surplus and By-Products to the Textile Industry

### 3.1. Textile Finishing

Whey proteins have been studied for their applicability as coatings in the textile industry (Table 3).

Cotton impregnation with whey protein isolate (WPI) has been shown to enhance staining with a natural dye rich in tannins. Adding proteins to the fabric makes it easy to color, due to the formation of insoluble complexes between proteins and tannins [39]. Casein, when used in the pre-treatment stages, also allowed the improvement of the dyeing process for natural dyes, especially for natural fibers like cotton and wool [30]. Milk is able to facilitate the dyeing process, not only because of its protein component [40]. The modification of textile dyes with lactose improved their water solubility, allowing the elimination of surfactants and mordants in the dyeing process [36].

Casein-based textile coatings to increase abrasion resistance and dye bonding have also been described. Through chemical modifications with acrylate esters and crosslinking agents, they can be used as an antistatic finish of natural (wool, cotton, silk) or synthetic (polyester) textile fibers [10].

Fabrics impregnated with milk proteins have also been explored, with the aim of increasing their thermal stability and flame retardation, due to the ability of this component to act as a water vapor absorber and as an oxygen barrier [31,32,33,41]. These can be applied in natural (cotton), synthetic (polyester) or mixed-textile fibers. In this study, it can be seen that the burning speed of the treated tissues decreases. In addition, although textile controls have superior thermal stability, the application of proteins helped to delay thermal degradation, which also led to a lower total loss of mass.

The antibacterial properties of some of the components of whey also been studied. Han et al. demonstrated that it was possible to improve the antibacterial properties of wool, obtaining 70% and 60% inhibition for *E. coli* and *S. aureus*, respectively, after the use of the crosslinker between microbial Transglutaminase and lactoferrin [34]. Transglutaminase was also used in a different approach. When used in conjugation with casein for the surface modification of wool, it resulted in an improvement in anti-felting properties and tensile strength [35].

### 3.2. Microencapsulation as Textile Finishing

As already mentioned, whey is rich in globular proteins that can be used as carriers for the micro/nanoencapsulation of bioactive compounds. This is a procedure that is already widely used in various sectors. It has the advantage of providing the possibility of the protection and controlled release of bioactive compounds, which can be used in the pharmaceutical or food sectors. On the other hand, this method can be easily applied in the textile industry, although few references are found in the literature on these applications. In the literature, there are only a few examples of textile applications of nanoparticles based on other milk proteins. Ghaheh et al. have studied the antioxidant effects of vitamin E that is encapsulated in BSA nanoparticles and applied onto cotton [42]. In this study, the nanoparticles were produced by ultrasonic emulsification, present a size between 200 and 300 nm and have the ability to encapsulate around 99% of the vitamin. The cotton fabrics present antioxidant activity and the finishing can resist up to 10 washing cycles [43]. In another study developed by Srisod and collaborators (2018), whey protein was used for the green synthesis of silver nanoparticles to improve antimicrobial properties in cotton textiles [38]. The use of whey microcapsules to encapsulate antimicrobial agents is very attractive in terms of the safety, biocompatibility, simplicity of synthesis and processing with antimicrobial efficiency.

In addition, the encapsulation of tetracycline, an antibiotic, was also studied using BSA and casein microspheres, which promotes an antibacterial coating for cotton and polyester fabrics [44]. These microcapsules not only demonstrated good encapsulation capacity, but also gave the textiles antimicrobial properties.

In industrial sectors other than textile, it is possible to find some milk protein microcapsule applications, namely from casein and whey protein, for microencapsulation of aromas [37]. The microcapsules may be produced using carboxymethylcellulose as an auxiliary. The use of carbohydrates as auxiliaries for the formation of protein microcapsules is quite common. Jain et al. studied β-carotene encapsulation using serum protein and gum arabic [45]. β-lactoglobulin also show an increase in self-aggregation properties, and sphere formation, after the addition of the same gum arabic [46]. Chen and Subirade also show that β-lactoglobulin also forms particles with chitosan [47], and this polysaccharide protects serum proteins from denaturation at temperatures up to 90 °C, due to their stabilizing effect [48]. Due to its globular structure, β-lactoglobulin can also aggregate with vitamins and bioactive compounds, such as folic acid. The β-lactoglobulin/folic acid complexes showing particle sizes below 10 nm showed stability over a wide range of pH values [49].

In relation to the other fractions of whey protein, due to their attractive properties and structure, they have been used as an encapsulating agent for bioactive substances, such as antibacterial, antifungals, antioxidants, among others, in order to develop new functional products [50,51,52,53].

## 4. Application of the Proteins Derived from the Milk By-Products to Other Industries

In addition to the textile applications previously presented as innovative solutions, casein and whey proteins have also been widely studied in other applications in several sectors besides food (Table 4). Despite its intrinsic relationship with the food industry, the applications have not only improved in terms of consumer products, but also in terms of the development of biomaterials for application in food packaging. On the other hand, the developments implicated in other applications have included nutraceutical, pharmaceutical, medicine and biomedical applications [54].

### 4.1. Packaging

In general, whey proteins have been extensively studied for food applications since the 1990s. They can be used in different forms, such as directly in liquid form, or treated and transformed into various food products, including powdered whey protein concentrates and isolates [55].

Caseins has been used directly in food industry not as by-product, but as an ingredient. However, in some dairy processes a surplus can exist, being recovered as a potential co-product. These proteins are usually used as ingredients in various food products to enhance their physical, functional and nutritional properties (e.g., foaming, thickening, emulsification, texture). They possess an excellent interaction with other molecules, stabilizing and emulsification properties and water-binding capacity [56]. Furthermore, caseins are inexpensive, non-toxic, and biodegradable and highly available [57].

Therefore, owing to their important biological properties and high encapsulation efficacy, caseins and casein micelles are used as a delivery system to ingredients and bioactive molecules in forms of micro- and nanoparticles [56]. Nowadays, technology has allowed for improvements to applications, and casein micelles may be useful as vehicles for the entrapment, protection and delivery of applications in other areas.

It is also useful to report the caseins and whey protein potential for use in edible film and coating production. Their good functional properties (high nutritional value, water solubility, and emulsification capability) make these constituents interesting for use in different applications such as coatings and food packaging [58]. Beyond from the packaging application, these edible films and coatings can be used as delivery systems. Food ingredients and bioactive molecules, such as flavours, antimicrobial agents, antioxidants, vitamins and minerals, can be incorporated into these films to provide a controlled rate and site of release.

However, casein films can be combined with other packaging materials for protecting products and improve their functionality [59]. Casein can also be used to produce insoluble films for the purpose of being used as a matrix for the immobilization of essential enzymes for industrial uses, such as glucose isomerase for the production of high-fructose corn syrup.

Mixed casein and whey films are widely varied and can be used to coat leather, paper and textiles, protecting them from moisture, too [60].

### 4.2. Biomedical Applications

Casein and whey protein have notably been used in biomedical applications in the medical and pharmaceutical area, as well as in tissue engineering.

Recently, there has been an increase in the number of studies that report the use of casein for nanofibers in several biomedical applications, such as in implants, tissue engineering scaffolding, wound healing, drug delivery systems, and so on [61,62]. These nanofibers were developed with casein, alone or in combination with other polymers such as polyvinyl alcohol (PVA) and polyethylene oxide (PEO). New technologies have also been enabling the incorporation of bioactive molecules and antimicrobial agents and increasing the impact on tissue engineering and wound healing.

In tissue engineering, casein has also been impactful due to its biocompatibility, biodegradability and higher availability, on scaffold developing and in binding materials as crosslinking agents for tissue engineering and biomedical applications [61]. Nowadays, casein hydrogels have been used for the delivery of bioactive compounds within nutraceuticals, pharmaceutics and cosmetics (hydrating and antiwrinkle agents) [61,63]. However, other forms, such as films, micelles, micro- and nanoparticles, can be used in drug and enzymatic delivery [57,61].

In recent years, many studies reported the use of whey proteins in the building blocks of nanoparticles, because of their biological properties (encapsulation efficiency for drugs, good stability and integrity, safety and lack of undesirable side effects), for applications in drug delivery [57].

Caseins have been explored extensively to produce biodegradable plastics. Studies have reported their mechanical properties, the strength of casein plastics, and similarities to bone, and so it has been suggested for use as a temporary bone graft that is absorbed by the body and promotes bone regeneration [60].

## 5. Conclusions and Future Remarks

The dairy industry produces many liquid byproducts from which whey proteins have been extracted, or milk surplus or losses from where casein is also obtained; those proteins have been investigated with the aim of recovery and application in the development of sustainable products. Due to their high value and accessible price, these proteins have been widely studied and applied, particularly in the food industry, due to their nutritional value, but also in other relevant industries where they have been creating innovative solutions. Some examples include coatings and materials for food packaging, new biomaterials and as bioactive carriers for nutraceutical, pharmaceutical and medicine applications, and in more recent innovation in the textile industry.

Accordingly, this concept of the symbiosis of the food sector with other industries responding to the current context of the circular economy must provide a possibility of developing eco-friendly and sustainable materials and will provide relevant insights to be adopted in non-food industries.

## Figures and Tables

**Table 1:** Main components and protein profile of bovine milk [3].

	Composition (g/L)	Molecular Weight (kDa)	Isoelectric Point
Lactose	48 ^(a)^		
Lipids	35–52 ^(a)^		
Salts	8–9 ^(a)^		
Proteins	35 ^(a)^		
_S1_-Casein	12–15	23.6	4.44–4.76
α_S2_-Casein	3–4	25.2	---
β-Casein	9–11	24.0	4.83–5.07
κ-Casein	2–4	19.0	5.3–5.8
β-Lactoglobulin	2–4	18.3	5.13
α-Lactalbumin	0.6–1.7	14.2	4.2–4.5
Serum albumin	0.4	66.4	4.7–4.9
Immunoglobulin	0.3–0.7	161.0 ^(b)^	5.5–6.8 ^(b)^
Lactoferrin	0.02–0.1	76.1	8.81
Lactoperoxidase	0.01–0.06 ^(c)^	77.5 ^(d)^	9.5 ^(d)^

^(a)^ [1]. ^(b)^ Value for the most abundant immunoglobulin in bovine milk, immunoglobulin G1, present at 0.3–0.6 g/L. ^(c)^ [4]. ^(d)^ [5].

**Table 2:** Sweet and acid whey powders composition (adapted, [12]).

	Sweet Whey Powder	Acid Whey Powder
Proteins	11.0–14.5%	11.0–13.5%
Lactose	63.0–75.0%	61.0–70.0%
Fat	1.0–1.5%	0.5–1.5%
Ash	8.2–8.8%	9.8–12.3%
Moisture	3.5–5.0%	3.5–5.0%

**Table 3:** Application of protein derived from the dairy by-products to the textile industry.

	Applicability	Technology	Results	References
**Textile finishing**	Textile coatings	Impregnation	- Enhance staining	[30]
			- Increase abrasion resistance	[10]
			- Increase thermal stability and flame retardancy	[31,32,33]
			- Improve antibacterial properties	[34]
			- Anti-felting properties and tensile strength	[35]
	Textile dyes	Chemical modification	- Improved water solubility, - Elimination of surfactants	[36]
	Functionalized textiles	Micro/nanocapsulation	- Improve antimicrobial properties	[37,38]
			Microencapsulation of aromas	[37]

**Table 4:** Application of protein derived from dairy by-products to several industries.

	Food Packaging	Biomedical
**Applicability**	Development of biodegradable materials for application in food packaging: e.g., edibles films and coatings production	- Biomedical applications, medical and pharmaceutical area, - Tissue engineering
**Technology**	- Micro/ nanoencapsulation - Impregnation - Extrusion	- Nanofibers, - Hidrogels, - Nanoparticles, - Tissue engineering scaffolding, - Wound healing, - Drug delivery systems
**Functions**	- Enhance physical, functional and nutritional properties - Improve applications as vehicles for entrapment, protection and delivery	- Incorporation of antimicrobial agents and other bioactive molecules

## Data Availability

Not applicable.
